# Kystes hydatiques de la rate: chirurgie radicale ou conservatrice?

**Published:** 2010-06-19

**Authors:** Abdelmalek Ousadden, Mohamed Raiss, Abdelmalek Hrora, Said AitLaalim, Mouna Alaoui, Farid Sabbah, Abdessalam Benamar, Mohamed Ahallat

**Affiliations:** 1 Clinique chirurgicale C, Hôpital Ibn Sina, CHU Ibn Sina, 10000 Rabat, Maroc

**Keywords:** Kyste hydatique, Rate, Splénectomie, Splénectomie partielle, Résection du dôme saillant

## Abstract

**Introduction:**

Concernant l’hydatidose, la localisation splénique vient en 3^ème^ position après le foie et les poumons. En l’absence de traitement médical réellement efficace, l’hydatidose splénique amène souvent à la chirurgie. L’apparition du traitement percutané et la tendance actuellement conservatrice de la chirurgie surtout pour une pathologie bénigne, remettent en cause la splénectomie radicale.

**Méthode:**

Notre travail rétrospectif, a porté sur 23 cas d’hydatidose splénique isolés ou multi-viscérale. Le diagnostic reposait principalement sur le couple échographie abdominale et sérologie hydatique. Ces patients ont bénéficié dans leur majorité d’une splénectomie ou d’une résection du dôme saillant du kyste.

**Résultats:**

La morbidité postopératoire a été plus importante en cas de résection du dôme saillant (une hémorragie d’origine splénique, 3 abcès sur cavité résiduelle et 2 récidives). La mortalité a été nulle. Nous proposons une classification qui permet de stratifier les indications opératoires en fonction du type de kyste hydatique de la rate.

**Conclusion:**

Le choix entre chirurgie radicale ou conservatrice reste difficile vu les contraintes de chacune, les complications postopératoires respectives et la diversité des situations cliniques. Notre classification facilite ce choix.

## Introduction

Le kyste hydatique (KH) est une infection parasitaire endémique dans de nombreux pays méditerranéens, entre autre au Maroc [1]. Par rapport aux autres localisations, la rate est rarement touchée (0.9 à 8.2%) [2-5]. Le KH constitue une des indications de la splénectomie en l’absence de traitement médical réellement efficace. Ce traitement chirurgical radical est remis en cause, d’abord par la tendance actuellement conservatrice de la chirurgie surtout pour une pathologie bénigne [2,3,6], ensuite par les connaissances anatomiques de la distribution vasculaire intrasplénique [7] et l’existence des traitements percutané et médical [8].

Par notre travail rétrospectif, nous comptons préciser les caractéristiques des kystes hydatiques de la rate (KHR) et proposons une classification facilitant le choix de la technique chirurgicale.

## Méthode

Notre étude a porté sur une série de 23 cas de KHR, colligés à la Clinique chirurgicale C de l’hôpital Ibn Sina de Rabat, entre 1981 et 2009. Nos malades se répartissaient en 11 femmes (48%) et 12 hommes (52%), avec un âge moyen de 38 ans et des extrêmes de 15 et 71 ans. L’origine rurale était prédominante.

Le délai entre le début de la symptomatologie et l’hospitalisation variait de 20 jours à 9 ans, le délai moyen étant de 18 mois. Dans leurs antécédents, 2 patients ont été opérés pour KH du foie, alors que 2 autres l’ont été pour KH pulmonaire. Chez 17 patients (73.9%) la douleur abdominale dominait la symptomatologie clinique. 14 malades (60.8%) présentaient une splénomégalie, alors que 4 avaient une hépatomégalie (17.4%). Chez 6 patients (26%), le KHR était découverte fortuitement dans le cadre du bilan d’une autre localisation hydatique. Le diagnostic a été fait grâce au couple échographie et sérologie hydatique.

L’échographie, pratiquée chez tous les patients, a confirmé la nature kystique et le siège splénique de la tumeur. Elle a permis de répartir ces kystes, dont 19 étaient uniques (82.6%), selon la classification de Gharbi. Ces KHR étaient de type I chez 8 patients (34.8%) dont 2 cas multiples, de type II chez un patient (4.3%), de type III chez 7 patients (30.5%) dont 2 cas multiples, de type IV chez un patient (4.3%) et de type V chez un patient (4.3%). Le type du KHR n’a pas été précisé chez 5 patients (21.8%). Le grand axe du KH mesurait de 6.7 à 21cm. L’échographie a aussi objectivé une hydronéphrose du rein gauche due à la compression par le KH dans un cas. La sérologie hydatique (Immunofluorescence et/ou réaction d’ELISA), pratiquée chez 18 patients, était positive 9 fois. Une hyper-éosinophilie de plus de 5% était retrouvée chez 3 des 23 patients (13%).

**Figure 1:**
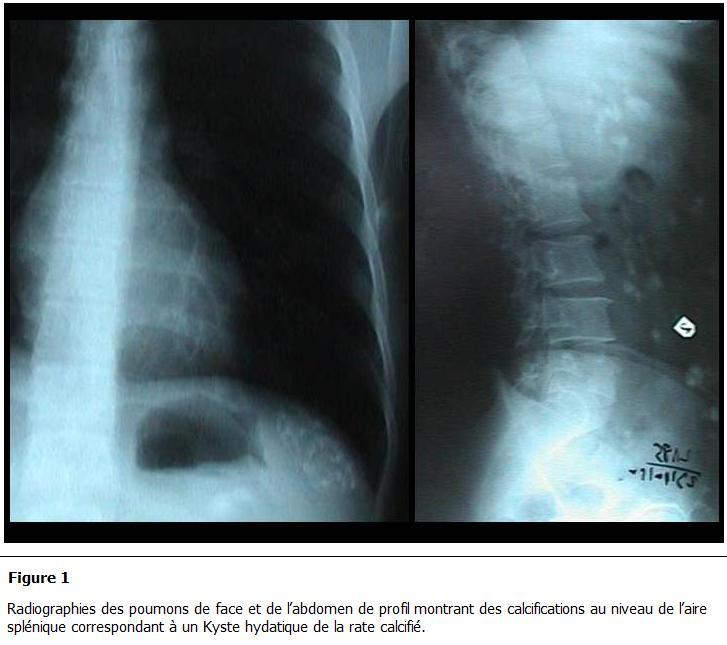
Radiographies des poumons de face et de l’abdomen de profil montrant des calcifications au niveau de l’aire splénique correspondant à un kyste hydatique de la rate calcifié

**Figure 2:**
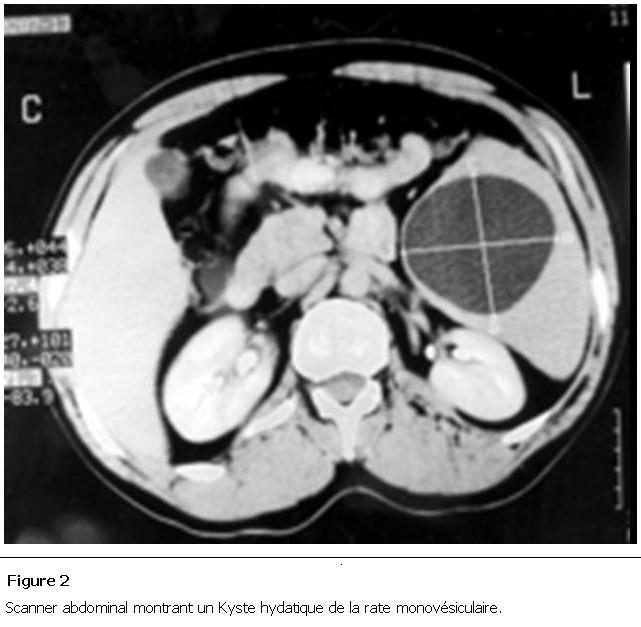
Scanner abdominal montrant un Kyste hydatique de la rate monovésiculaire

**Figure 3:**
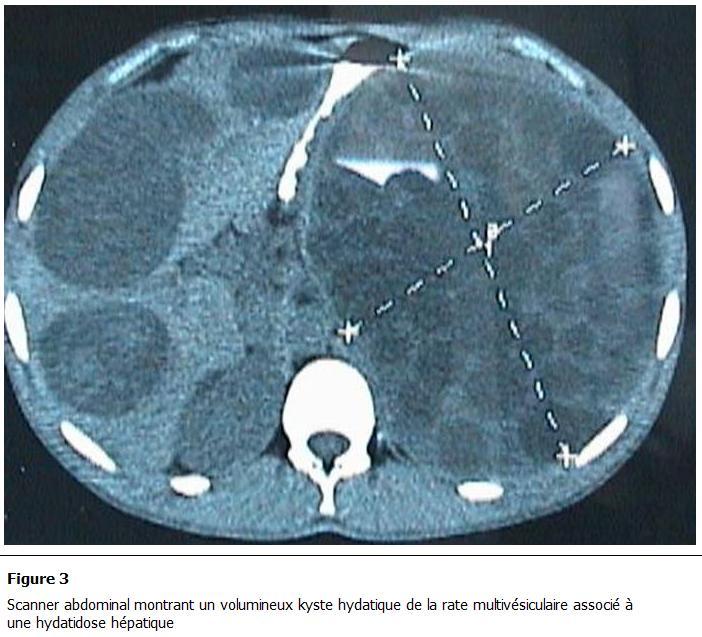
Scanner abdominal montrant un volumineux kyste hydatique de la rate multivésiculaire associé à une hydatidose hépatique

**Figure 4:**
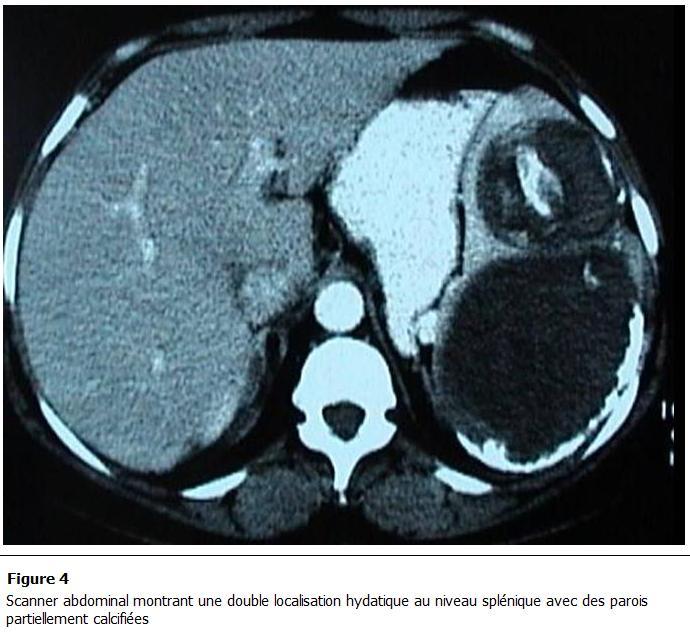
Scanner abdominal montrant une double localisation hydatique au niveau splénique avec des parois partiellement calcifiées

**Figure 5:**
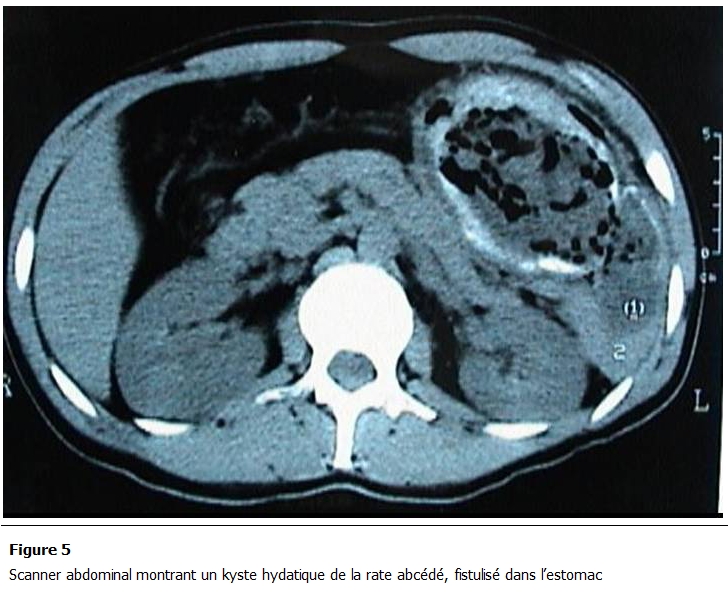
Scanner abdominal montrant un kyste hydatique de la rate abcédé, fistulisé dans l’estomac

Parmi les autres examens pratiqués, la radiographie pulmonaire et le cliché d’abdomen sans préparation montraient des calcifications se projetant sur l’aire splénique dans 2 cas (Figure 1), une surélévation de la coupole diaphragmatique gauche dans 7 cas et dans 3 cas une hydatidose pulmonaire associée. Le scanner abdominal (Figure 2, 3, 4, 5) était réalisé chez 7 patients pour explorer les rapports et extensions du kyste. En définitive, le KHR était isolé chez 12 patients (52.2%). Chez 10 patients (43.5%), il était associé à une localisation hydatique hépatique (Figure 3), à laquelle s’associait dans 3 cas (13%) une localisation péritonéale, dans un cas une localisation rétropéritonéale (4.3%), et dans un autre une localisation pulmonaire (4.3%).

Chez 2 patients, une localisation pulmonaire (8.7%), s’associait seule à la localisation splénique. Sur les 23 patients, seul un n’a pas été opéré. Son hydatidose multiviscérale a été traité médicalement (Albendazole).

**Figure 6:**
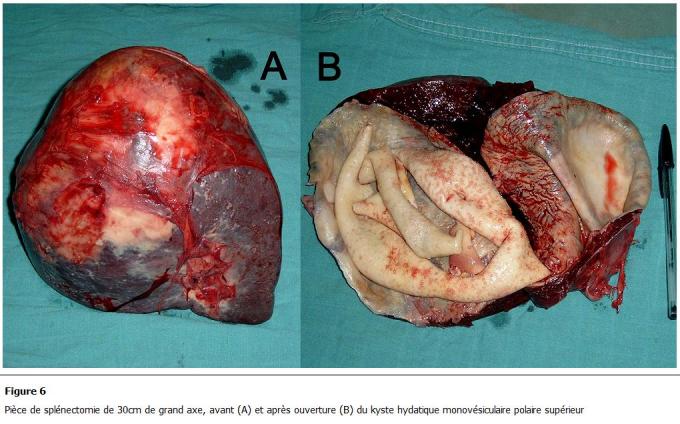
Pièce de splénectomie de 30cm de grand axe, avant (A) et après ouverture (B) du kyste hydatique monovésiculaire polaire supérieur

**Figure 7:**
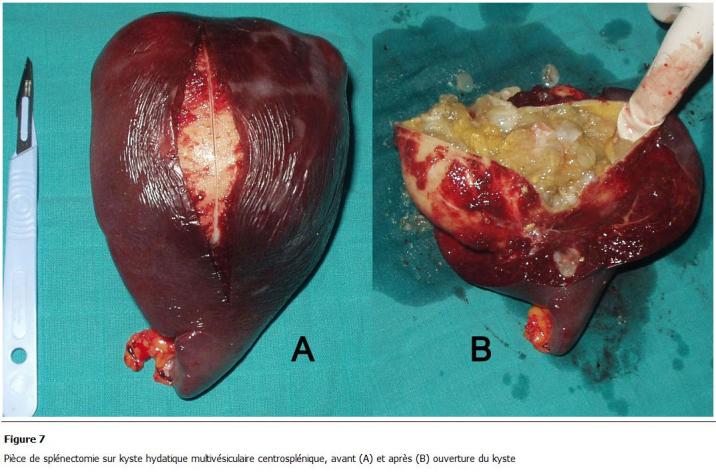
Pièce de splénectomie sur kyste hydatique multivésiculaire centrosplénique, avant (A) et après (B) ouverture du kyste

La voie d’abord était médiane 17 fois (77.3%), bi-sous-costale une fois (4.5%), sous-costale gauche 3 fois (13.7%), sous-costale droite une fois (4.5%). Aucun cas n’a été opéré par cœlio-chirurgie. Les kystes spléniques retrouvés étaient de localisation polaire supérieure (Figure 6) dans 7 cas (31.8%), polaire inférieure dans 4 cas (18.2%), médiosplénique (Figure 7) dans 3 cas (13.6%), de la face externe dans 2 cas (9.1%), occupant toute la rate dans 2 cas (9.2%) et multiples dans 4 cas (18.2%). Chez les 22 patients opérés, le KH splénique a motivé la réalisation de 9 splénectomies totales (40.9%) (Figure 6, 7), 11 résections du dôme saillant (RDS) (50%) et une splénectomie partielle (4.5%).

Chez une patiente, le kyste hydatique centrosplénique calcifié a été respecté (4.5%) alors que les KH hépatiques associés ont été traité. Les gestes associés à la cure du KHR ont été les cures des autres localisations hydatiques hépatiques et péritonéales associées, une cholécystectomie ainsi que l’aveuglement de 2 fistules biliaires, d’une fistule kysto-gastrique (Figure 5) et d’une autre kysto-colique. La vaccination anti-pneumococcique a été faite chez tous les patients ayant bénéficié d’une splénectomie. Un traitement médical antiparasitaire à base d’albendazole a été prescrit en postopératoire, pour une durée de 6 mois chez 2 patients présentant un hydatidose multi-viscérale.

## Résultats

Dans notre série, la mortalité a été nulle. Les suites opératoires immédiates étaient marquées dans le groupe des 9 splénectomisés, par une hyperthermie isolée révolutive sous antibiothérapie chez 3 patients (33.3%), et par une pneumopathie banale chez un patient (11.1%). Dans ce groupe, le séjour hospitalier a varié de 19 à 23 jours, alors que dans le groupe des 11 patients ayant bénéficié d’une RDS du KH splénique, le séjour a été plus long (23 à 45 jours). Dans ce dernier groupe, une hémorragie importante d’origine splénique a été notée dans 1 cas (9%), alors qu’une abcédation de la cavité résiduelle a été constaté 3 cas (27.3%). Cette abcédation est apparue chez le premier patient, au 10^ème^ jour postopératoire, traitée avec succès par une antibiothérapie associée à un drainage écho-guidé percutané. L’abcédation apparue dans le deuxième cas, au 5^ème^ mois postopératoire, a bénéficié d’un drainage échoguidé percutané associé à une antibiothérapie, ce qui a permis une guérison première. Puis une récidive de l’abcès a conduit à un drainage chirurgical 9 mois après la première intervention. Une deuxième récidive de l’abcès 2 mois après, a imposé la splénectomie. L’abcédation du 3^ème^ cas, atteignant 20 centimètres de grand axe, a été diagnostiqué 2 ans après l’intervention et a été traité par complément de RDS avec drainage chirurgical.

Les suites opératoires ont été simples pour les autres cas, en notant qu’il y a eu deux récidives hydatiques suite à une RDS (18.2%) de KHR. La première au niveau splénique, alors que la seconde était dans l’arrière cavité des épiploons.

**Tableau 1:** Classification des kystes hydatiques de la rate et traitement chirurgical correspondant

**Type**	**Caractéristiques**	**Techniques proposées**
*Type A*	KHR unipolaire supérieur ou inférieur, épargnant plus du quart de la rate	La splénectomie partielle préférable à la RDS
*Type B*	KHR non unipolaire, épargnant plus du quart de la rate	La RDS préférable à la splénectomie totale
*Type C*	KHR détruisant plus de trois quarts de la rate	La splénectomie totale est préférable à la RDS

KHR : Kyste Hydatique de la rate, RDS : résections du dôme saillant

## Discussion

L’hydatidose est une helminthiase provoquée par le développement chez l’homme de la forme larvaire de l’echinococcus granulosus. L’hôte définitif est le plus souvent le chien [1]. L’hôte intermédiaire, contaminé par voie digestive, est le plus souvent le mouton et accidentellement l’homme [1]. L’embryon traverse alors la paroi intestinale, gagne le foie (1^er^ filtre), par la veine porte pour s’y localiser ou pour gagner les poumons (2^ème^ filtre) par voie cave, puis n’importe quel autre organe par l’intermédiaire de la circulation systémique. Le KH est ainsi localisé le plus souvent dans le foie puis dans les poumons [1]. La localisation splénique vient en 3^ème^ position. D’autres voies d’atteinte splénique ont été évoquées : l’atteinte par contiguïté (trans-pariétale gastrique ou colique) la voie lymphatique et la voie veineuse porto-splénique rétrograde [4]. Ces hypothèses physiopathologiques pourraient expliquer le nombre important de KHR isolé (52% dans notre série).

Le KHR touche surtout les adultes, vers 30 à 40 ans, avec une légère prédominance féminine [2-5]. Dans 20 à 62.5% des cas, le KHR est associé à d’autres localisations hydatiques, en particulier hépatique ou péritonéale [4,9]. Cela intéresse 48% des cas de notre série. Le développement du KHR est pauci symptomatique et lent avec une phase de latence clinique de 2 à 20 ans [2-5]. Les motifs de consultation les plus fréquents sont la douleur suivi par la constatation d’une masse de l’hypochondre gauche (ou d’une splénomégalie) et la découverte fortuite [2,3]. L’abcédation, la fissuration avec anaphylaxie et la rupture dans la plèvre, l’estomac, le colon ou à la peau sont les complications qui peuvent révéler un KHR. L’échographie, le scanner et l’imagerie par résonance magnétique de l’abdomen sont les examens les plus utiles au diagnostic [4]. Ils sont associés à la sérologie hydatique pour une plus grande certitude diagnostic. Ces examens permettent aussi le diagnostic des formes asymptomatiques et polyviscérales [2].

La stadification échographique du KH du foie de Gharbi [10] est utilisée par analogie au KHR [2]. Les radiographies de l’abdomen sans préparation et des poumons ont valeur d’orientation. Elles peuvent objectiver une calcification ou une masse de tonalité hydrique qui se projette dans l’aire pulmonaire ou splénique. L’hyper-éosinophilie, peu retrouvée dans notre série, est non spécifique.

La prévention repose sur l’éducation sanitaire des populations, le contrôle vétérinaire de l’abattage du bétail, l’abattage des chiens errants avec le recensement et le vermifugeage des chiens domestiques. Le traitement médical par Albendazole ou Mébendazole, malgré des résultats insuffisants, est utilisé pour les formes multi-viscérales et en tant qu’adjuvant aux autres traitements [7]. Il a été prescrit chez 3 de nos patients, 2 fois associé à la chirurgie. Celle-ci, est le traitement le plus efficace [11,12], même si la ponction-aspiration écho-guidée des kystes de type I et II avec injection de produit sclérosant ait été proposée [8].

La splénectomie totale a l’avantage de supprimer l’organe parasité, d’éviter les récidives (2 cas dans le groupe des RDS de notre série) et les complications liées à la coque résiduelle [2] (3 cas dans notre série). Mais elle est de réalisation délicate en cas d’adhérences kysto-viscélales. Sa mortalité va de 3.7 à 22.5%, alors que sa morbidité propre est de 21 à 25% [8] (Respectivement 0% et 44.4% pour notre série). Des complications graves peuvent être rencontrées, telles que le choc hémorragique, l’abcès sous phrénique ou les accidents infectieux (pneumopathies fulminantes, septicémies…) surtout chez l’enfant [14].

A long terme, le développement de lésions d’athérome, amenant à l’ischémie myocardique, pourrait être favorisé [6]. Dés lors, la splénectomie totale parait moins légitime surtout en présence d’une pathologie bénigne. Cela doit amener à réserver cette technique aux kystes multiples, à ceux siégeant sur une rate pathologique, aux kystes hilaires et intra-parenchymateux centraux chez qui la résection du dôme saillant représenterait un risque hémorragique important, et en cas d’adhérences kysto-viscérales multiples. Les techniques chirurgicales conservatrices sont donc à encourager bien qu’elles aient des complications propres. Ces techniques sont la marsupialisation actuellement délaissée, la périkystéctomie limitée ou RDS et la splénectomie partielle qui repose sur la connaissance parfaite de la segmentation vasculaire splénique et sur l’amélioration des matériels de sutures et d’hémostase [4,7].

La RDS a l’avantage d’être une intervention bénigne, peu hémorragique, presque toujours réalisable, dès lors que le KH est accessible à la surface de la rate [4,5]. Son inconvénient est de laisser du périkyste en place qui peut être siège de cavité résiduelle et d’infection postopératoire [2,4] (27.3% dans notre série). La splénectomie partielle, apparaît comme une attitude intermédiaire qui évite les complications de la RDS et de la splénectomie totale en conservant plus de 25% du parenchyme splénique [14].

Le choix de la voie d’abord dépend aussi bien de la localisation du ou des kystes spléniques, que de l’association à d’autre localisation kystiques hépatiques, péritonéales, ou autres, sans oublier le type de ces kystes et l’existence d’éventuelles complications. L’abord laparoscopique est réalisable, à basse pression, pour presque tous les cas, avec de bon résultats à court et à long terme [1,11,15].

A la lumière de notre travail, nous avons subdivisé les KHR en 3 types et stratifié les indications thérapeutiques (Tableau 1). Le type A correspond au KHR unipolaire supérieur ou inférieur, épargnant plus du quart de la rate, chez qui la splénectomie partielle serait préférable à la RDS. Le type B correspond au KHR non unipolaire, épargnant plus du quart de la rate, chez qui la RDS serait préférable à la splénectomie totale. Le type C correspond KHR détruisant plus de trois quarts de la rate, chez qui la splénectomie totale est préférable à la RDS.

## Conclusion

Le choix de la technique chirurgicale en cas d’hydatidose splénique est délicat quand on met en balance les complications postopératoires des résections du dôme saillant et les risques à long terme de la splénectomie radicale. La splénectomie partielle pourrait être le compromis idéal quand elle est réalisable. La cœlioscopie serait un autre pas vers la diminution de la morbidité thérapeutique. Notre classification permet de mieux choisir la technique opératoire en tenant compte de la nature du kyste, de sa localisation et des contraintes relatives à chaque technique.
